# Patient experiences and perceptions of kidney stone surgery: what lessons can be learned from TikTok?

**DOI:** 10.3389/fsurg.2024.1374851

**Published:** 2024-03-20

**Authors:** Patrick Juliebø-Jones, Lazaros Tzelves, Christian Beisland, Ingunn Roth, Bhaskar K. Somani

**Affiliations:** ^1^ EAU YAU Endourology Group, Arnhem, Netherlands; ^2^ Department of Urology, Haukeland University Hospital, Bergen, Norway; ^3^ Department of Clinical Medicine, University of Bergen, Bergen, Norway; ^4^ Second Department of Urology, National and Kapodistrian University of Athens, Sismanogleio General Hospital, Athens, Greece; ^5^ Department of Urology, University Hospital Southampton, Southampton, United Kingdom

**Keywords:** urolithiasis, ureteroscopy, TikTok, social media, shockwave lithotripsy

## Abstract

**Introduction:**

The aim of this study was to perform an evaluation of patient experiences and perceptions regarding kidney stone surgery on the social media platform TikTok. An increasing number of the public use social media (SoMe) as a platform to share their views regarding their experiences related to surgical treatment.

**Methods:**

Using the hashtag #kidneystonesurgery, the 100 most recent video posts as of 01.01.2024 on TikTok were included. As well as demographic data such as gender and location, thematic content was also collected. To achieve this, a previously published framework was used and adapted for application in the setting of kidney stone surgery. This was piloted on 20 sample videos to assess its feasibility before revision and establishment of the final framework. This included the following key areas: Pain, Complications, Anxiety, Recovery, Return to work, Finances, Treatment delays, Diet and Prevention and stent complaints.

**Results:**

The majority of posts (95%) were from North America, 80% by females and the mean number of video views was 92,826 (range: 261–2,000,000). 76% of the videos discussed ureteroscopy (URS). 49% were filmed at the hospital, which was named in 9% of the videos. Top three topics discussed were: Recovery (65%), pain (62%) and stents (55%). This was followed by anxiety (39%) and complications (24%). 12% of these videos uploaded by lay people included basic medical information that was wholly incorrect. More than half of the posts (51%) were negative in tone. Treatment delays (5%) and a lack of sufficient preoperative information (4%) were also raised, that appeared to contribute to the negative reports. However, the main cause for negative tone owed to the 80% of the patients (*n* = 44) who discussed stents that focused their video on the pain suffered from the post operative stent.

**Conclusion:**

There is a high level of usership and engagement on TikTok on the subject of kidney stone surgery. The proportion of negative videos is high and much of this is related to the bothersome stent symptoms and complications. This could easily lead to misperceptions among potential patients about the true burden of such adverse events.

## Introduction

With an increasing prevalence of kidney stone disease (KSD) worldwide, the volume of surgeries performed has increased accordingly (1, 2). Traditional outcome measures of interest have been largely focused on objective parameters such as stone free rate and complications. However, in the recent era, patient experience and the impact on quality of life related to surgery has been appreciated more (3–5). Research across a number of surgical fields has highlighted how patients use social media (SoMe) as a platform to share their views regarding their experiences related to surgical treatment (6, 7). At the same time, patients awaiting surgery often wish to hear first-hand, the experiences of others and SoMe can allow for this. In a survey of patients undergoing maxillofacial surgery, it was found that SoMe can influence decision to undergo surgery as well as by which medical provider (8).

While there are a number of SoMe platforms available, TikTok, which has over one billion active users per month, is one which allows for users to make extended videos (maximum 10 min) (9). These are often in a talking heads style where users discuss a topic, and in this way, it lends itself to recounting their own treatment and experiences as a patient. While SoMe findings related to patient experience has been studied in a number of other surgical fields, there is a very limited amount related to the management of KSD. An increasing proportion of the lay community use the internet and SoMe to learn more about their health-related problems (10). This has only been further augmented as a result of the Covid-19 pandemic.

Our aim was to perform an evaluation of patient experiences, perceptions and lessons learnt from kidney stone surgery on TikTok.

## Materials and methods

After creating an anonymous account, a search was performed on the SoMe platform TikTok using the hashtag #kidneystonesurgery. The 100 most recent video posts as of 01.01.2024 were included. Videos considered eligible were those in the English language, uploaded by self-identifying patients, and videos deemed to be made for the purposes of humour e.g., comedy sketches were excluded. Given that all data was freely available in the public domain, it was determined that ethical approval was not required. Content uploaded from minors was excluded. As well as demographic data such as gender and location, thematic content was also collected. To achieve this, a previously published framework was used and adapted for application in the setting of kidney stone surgery (6). This was piloted on 20 sample videos to assess its feasibility before revision and establishment of the final framework. To this end, data was collected on the following key areas: (1) Pain (2) Complications (3) Anxiety (4) Recovery (5) Return to work (6) Finances (7) Treatment delays (8) Diet and Prevention (9) Stent (10) Gratitude to healthcare workers and (11) Activities of daily life (ADLs). Supplementary data was also collected in the form of where filming occurred, and timing in relation to surgery among other characteristics. It was also noted if the user stated any basic medical facts, which were completely incorrect.

## Results

Of the 100 videos evaluated, 95% of posts were uploaded from North America and 80% by females. The mean number of video views was 92,826 (range: 261–2,000,000) while the mean number of likes and comments was 7,669 (range: 2–208,200) and 113 (range: 0–2,366), respectively (Table 1). Elective surgery was the most common setting (64%). 76% of the videos discussed ureteroscopy (URS) and most were captured after the surgery had been performed. 49% were filmed at the hospital, which was specifically named in 9% of the videos. 10% showed the viewers their removed stent and 2% showed their own radiographic images.

**Table 1:** Summary of demographics.

Mean number of views (range)	92,826 (261–2,000,000)
Mean number of likes (range)	7,669 (2–208,200)
Mean number of comments (range)	113 (0–2,366)
Mean number of times video set as a favourite (range)	249 (0–8,599)
Country of video origin	
North America	95%
Asia	3%
Europe	2%
Gender	
Male	20%
Female	80%
Setting	
Elective surgery	64%
Emergency surgery	14%
Unknown	22%
Surgery type	
URS	76%
PCNL	3%
SWL	2%
Unknown	19%
Timing	
Before surgery	20%
After surgery	64%
Before and after surgery	16%
Filmed at hospital	49%
Hospital named	9%

URS, ureteroscopy; PCNL, percutaneous nephrolithotomy; SWL, shockwave lithotripsy.

The top three topics discussed were: Recovery (65%), pain (62%) and stents (55%). This was followed by anxiety (39%) complications (24%) and ADLs (22%) (Table 2). All the complications involved readmission to the emergency department. 12% of these videos uploaded by lay people included basic medical information that was wholly incorrect. These covered how the surgery was performed, potential complications and evidence supporting natural remedies. None of the videos discussed surgical technology. Discussion of stone diet (5%) and finances (3%) were relatively low. 5% of the patients reported that the clinician had told them about being completely stone free on leaving the hospital.

**Table 2:** Summary of content analysis.

Domains covered	
Recovery	65%
Pain	62%
Stent	55%
Anxiety	39%
Complications	24%
Activities of daily life	22%
Disease recurrence	14%
Need for multiple surgeries	24%
Incorrect basic medical information:	12%
Return to work	7%
Diet and stone prevention	5%
Treatment delays	5%
Lack of sufficient pre-operative information	4%
Gratitude to healthcare providers	4%
Financial costs	3%
Technology in stone surgery	Zero
Tone	
Positive	22%
Neutral	37%
Negative	51%

More than half of the posts (51%) were negative in tone. Treatment delays (5%) and a lack of sufficient preoperative information (4%) were also raised, that appeared to contribute to the negative reports. However, the main cause for negative tone owed to the 80% of the patients (*n* = 44) who discussed stents that focused their video on the pain suffered from the post operative stent.

## Discussion

This study highlights that patients do use SoMe platforms such as TikTok to communicate their patient experiences related to kidney stone surgery. As also seen by the volume of comments, patients also use this as a vehicle to communicate with other patients regarding all areas of the treatment pathway. The findings also serve as a reminder to clinicians that patients may well film while at the hospital, both during elective and emergency admissions, as well as potentially name the medical provider publicly. The representation of complications related to kidney stone surgery could easily give an impression to a lay person that the true complication burden is much higher than what is formally reported in studies. Doctors should therefore counsel patients that patient experiences on social media should be taken with caution. This is highly relevant given the findings of Kunitsky et al. where it was found that 51% of respondents in a survey answered that they use a combination of Reddit, Facebook and/or YouTube to gain medical information (11). Stents are well recognised to be associated with negative quality of life in some patients (12). This study confirms this as an issue and supports the supposition that pain related to indwelling stent is an issue that surgeons should proactively take up with patients pre-operatively. Videos uploaded on the topic of kidney stones also originate from health care professionals. In a recent study by Diaz et al, which evaluated the educational content of such videos, the overall scores were quite low (13). More attention towards creating educational content at the appropriate level is needed. Yilmaz et al. assessed similar YouTube content that focused on miniaturised PCNL and found that these seemed to be aimed at other medical professionals than patients (14). Moving away from SoMe, the readability of educational materials found online have also been found to be substandard (15). Assessment of the online education content produced by the European Association of Urology (EAU) has also been recently performed (16). This study found that while the readability of this web-based content is superior to the abovementioned sources, further simplification is much needed. In recent times, there has been an increased demand by some journals for scientific manuscripts to also include a patient summary. While the possible merits of this are clear, a recent analysis of 266 articles by Ganjavi et al. found these also to be too difficult to the lay community to read (17). Such are the advances that have taken place, much attention is given by urologists on SoMe to new technologies such as novel laser platforms (e.g., Thulium fiber laser) and new accessories (e.g., suction access sheaths) (18, 19). It is interesting but perhaps not surprising that none of this was ever mentioned by patients. There are many areas that health care professionals need to stay up to date on such as artificial intelligence and new simulation methods, staying up to date with social media and its impact on health care is yet another new field (20).

This study does have certain limitations. Only 100 videos were sampled and these were the most recent videos captured. Inclusion of older videos may have provided a better impression of overall viewership and engagement. Sampling of more videos would have allowed for the findings to be more generalisable. This study only evaluated videos in the English language and may therefore misrepresent findings on a worldwide level. Different surgical interventions were also not differentiated in the analysis. However, the merits of this novel study include that it assesses the patient perspective as opposed to the content posted by health care professionals (13). Given the relative low volume of research focused on quality of life compared to those evaluating objective outcomes, studies such as this one that offer a new means to gauge patient experiences are arguably welcomed (3). More prospective studies of a qualitative nature are needed to explore patient experiences and perceptions of kidney stone surgery. This would allow the domains highlighted in this study to be explored more substantially.

## Conclusion

There appears a high level of usership and engagement on TikTok on the subject of kidney stone surgery. Much of this is filmed by patients while physically being at the hospital site. In this way, it is being used as a platform to share and communicate experiences among patients. The proportion of negative videos is quite high and much of this is related to the bothersome stent symptoms and complications. This could easily lead to misperceptions among potential patients about the true burden of such adverse events. This supports the need for comprehensive pre-operative counselling.

## Data Availability

The raw data supporting the conclusions of this article will be made available by the authors upon reasonable request.
